# Predicting DNA Methylation State of CpG Dinucleotide Using Genome Topological Features and Deep Networks

**DOI:** 10.1038/srep19598

**Published:** 2016-01-22

**Authors:** Yiheng Wang, Tong Liu, Dong Xu, Huidong Shi, Chaoyang Zhang, Yin-Yuan Mo, Zheng Wang

**Affiliations:** 1 School of Computing, University of Southern Mississippi, 118 College Drive #5106, Hattiesburg, MS 39406, USA; 2 Department of Computer Science and Christopher S. Bond Life Sciences Center, University of Missouri, 201 Engineering Building West, Columbia, MO 65211, USA; 3 Department of Biochemistry and Molecular Biology, Georgia Regents University, 1120 15^th^ Street, Augusta, GA 30912, USA; 4 Department of Pharmacology and Toxicology, University of Mississippi Medical Center, 2500 North State Street, Jackson, MS 39216, USA

## Abstract

The hypo- or hyper-methylation of the human genome is one of the epigenetic features of leukemia. However, experimental approaches have only determined the methylation state of a small portion of the human genome. We developed deep learning based (stacked denoising autoencoders, or SdAs) software named “DeepMethyl” to predict the methylation state of DNA CpG dinucleotides using features inferred from three-dimensional genome topology (based on Hi-C) and DNA sequence patterns. We used the experimental data from immortalised myelogenous leukemia (K562) and healthy lymphoblastoid (GM12878) cell lines to train the learning models and assess prediction performance. We have tested various SdA architectures with different configurations of hidden layer(s) and amount of pre-training data and compared the performance of deep networks relative to support vector machines (SVMs). Using the methylation states of sequentially neighboring regions as one of the learning features, an SdA achieved a blind test accuracy of 89.7% for GM12878 and 88.6% for K562. When the methylation states of sequentially neighboring regions are unknown, the accuracies are 84.82% for GM12878 and 72.01% for K562. We also analyzed the contribution of genome topological features inferred from Hi-C. DeepMethyl can be accessed at http://dna.cs.usm.edu/deepmethyl/.

DNA methylation represents the addition of a methyl group to the fifth carbon of the cytosine or adenine^1^. DNA methylation occurs more frequently at CpG sites, where a guanine nucleotide follows a cytosine nucleotide in the sequence of the genome^2^,^3^. In some regions, the frequency of CpG sites is up to 10 times greater than the average. These regions are called CpG islands (CGIs)^4^. CpG islands have a GC percentage greater than 50% with at least 200 base pairs long. Generally speaking, CpG sites outside the CGIs are mostly methylated, whereas CpG sites within CGIs are mostly unmethylated^5^. This difference indicates that CGIs usually have distinguished patterns of methylation, which may be important in gene regulation or gene mutation^6^,^7^.

DNA methylation has been found to have influences on the expression of genes and functional regulation of proteins^8^,^9^. According to recent studies^10^,^11^,^12^, DNA methylation can affect the onset and progress of various cancers and complex diseases. There are more methylated promoters and suppressors found in abnormal cell lines^13^. The aberrance of DNA methylation is one of the typical features of cancers such as acute myeloid leukemia^14^. However, the mechanistic link between aberrance of DNA methylation and leukemia is not well understood. Recent studies investigated DNA methylation in various cancers such as breast cancer^15^,^16^. The results indicate that abnormal DNA methylation usually occurs at some specific genomic locations^17^,^18^.

Recent advances in methylation sequencing technologies allow the identification of genome-wide methylated sites in DNA^19^,^20^. One way of profiling methylation patterns of DNA is via the use of bisulfite treatment of DNA followed by next-generation sequencing, which is known as bisulfite sequencing^21^. The current bisulfite sequencing methods include whole-genome bisulfite sequencing (WGBS)^22^ and reduced representation bisulfite sequencing (RRBS)^23^. Comparing to WGBS, RRBS reduces the amount of sequencing by using representative fractions of the genome. Therefore, RRBS specifically profiles and analyzes the methylation pattern for the regions with a high CpG content^24^.

Methods have been developed to predict the methylation states at CpG sites, windows or segments of a genome^25^,^26^,^27^,^28^. Most of the current methods for methylation prediction assume that the methylation states are binary classes, that is, a CpG site or a window is either methylated or un-methylated (methylation-resistant)^29^. However, some other methods classified the methylation level to multiple classes^30^. Among these methods, predictions were usually limited to specific regions such as CGIs^28^,^31^. Predictive features used by these methods included DNA composition^32^, GC content^28^, sequence patterns^33^, and methylation state of neighboring region^30^. Recent methods also used pseudo nucleotide composition to predict the methylations sites of a genome^32^,^34^. The DNA composition and methylation state of sequential neighbors are the two most common features among these methods^30^,^33^.

One of the features that have not been used in predicting DNA methylation is chromosome interaction. The Hi-C technique enables the investigation of both intra- and inter-chromosomal contacts in a genome^35^. The analysis of the genome at 1–1000 kilo-base resolution captures the overall genome spatial conformational arrangements. The 1 kilo-base resolution would further capture the contacts between the genes within the genome^36^. The Hi-C experiments cut the crosslink DNA with restriction enzyme and ligate them under extremely dilute conditions that favor intermolecular ligation. The experiments then purify and shear the ligated DNA segments to obtain paired-end reads. The paired-end Hi-C reads are mapped to the reference genome. After mapping, the data are binned and normalized into the Hi-C contacts library, which indicates certain positions are spatially close in the three-dimensional space.

Although many methods have been developed to predict the methylation state of specific regions, the prediction of the methylation state of CpG sites in the loci of long non-coding RNAs (lncRNA) has received little attention. LncRNA are transcripts of non-coding genes ranging from 200 bases to 100 kilo-bases (kb)^37^, yet their potential activities in human diseases have not been significantly unveiled. Recent studies on gene expression indicate that lncRNA may function as the connector between DNA and specific chromatin remodeling activities^38^, and the expression level of lncRNA usually is lower than the ones of protein-coding genes^39^. Furthermore, lncRNA expression might be a main factor in carcinogenesis^40^. The exact mechanism of how lncRNAs influence cancer is unknown, but abnormal lncRNA expression may be a factor causing cancer by affecting major genetic processes. We evaluated our methylation predictions on the CpG sites in lncRNA loci.

In this study, we applied a deep learning algorithm, stacked denoising autoencoders (SdAs), to predict DNA methylation status of CpG sites. Different from traditional learning algorithms, the training of SdAs contains two stages: an unsupervised pre-training stage using unlabeled training data and a supervised fine-tuning stage using labeled data (data with known target values). We used sequential features generated within a window of the genome and features generated from the three-dimensional topology of a genome indicated by the Hi-C experiment^41^. We did extensive tests of our method through several benchmarks. In the first benchmark (Benchmark 1), we included the methylation level of sequential neighboring regions as features, whereas in the second benchmark (Benchmark 2), we excluded this type of features to increase prediction difficulty. We also benchmarked the influences of unlabeled data in deep learning and the influences of the genome topological features on the prediction accuracy.

## Results

### Overview

We built SVM and SdA models to predict binary DNA methylation status of CpG sites (methylated or unmethylated). We applied our predictive models on lymphoblastic cell lines (GM12878) and chronic myelogenous leukemia cell lines (K562) to compare the performance of predictions on the healthy and cancer cell lines. Two types of windows were defined to generate features from sequentially and topologically neighboring regions of the genome (details see Methods). To better understand the factors influencing the performance of SdAs, we applied different amounts of unlabeled pre-training samples, hidden layers, numbers of denoising autoencoders in each layer, and pre-training and training epochs. We also tested the performance of predicting methylation states of the CpG sites in lncRNAs loci.

In Benchmark 1, we measured the performance of our predictors using the metrics accuracy (Acc), specificity (Sp), sensitivity (Se), Matthews’s correlation coefficient (MCC) and Receiver Operating Characteristic (ROC) curve using leave-one-out cross-validation. For the blind test data, only test accuracy was applied to evaluate the performance of SdA and SVM models. Different window-A sizes of each target CpG site, from 500–1000 nt, were tested. The definition of window-A can be found in the Methods section.

Moreover, we conducted Benchmark 2 by eliminating the features containing the methylation state of sequential neighboring regions but only using features of (1) the methylation level of three-dimensional (3D) neighboring regions and (2) sequential composition patterns of the DNA sequence. In order to find the impact of the Hi-C based (3D genome topology) features, we tested the performance of using features generated from randomly selected windows that do not have any Hi-C contact to the target region. Different “Hi-C ranges” (from 10 K to 50 K) were used to benchmark the impact of including different amounts of Hi-C window-B (definition see Methods) features. In addition, we performed a blind test by randomly combining the samples from chromosome 1 with the ones from chromosome 21.

### Chromosome-wide analysis of methylation patterns and preparing training and testing data sets

**Figure 1:**
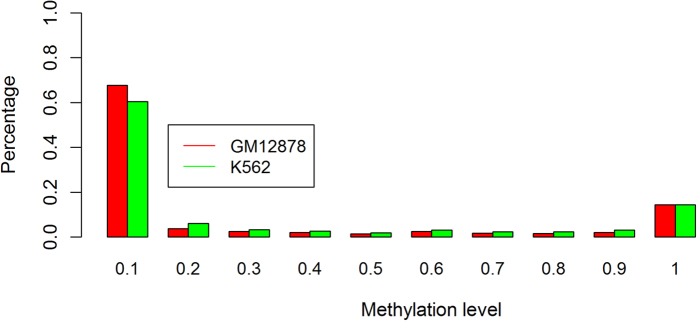
Distribution of DNA methylation levels on CpG sites for chromosomes 1, 2 and 3 for GM12878 and K562.

The methylated and unmethylated samples were defined based on parameters α and β (details see Methods). In order to balance our training dataset, we examined the distribution of PercentMeth (explained in Methods) values from RRBS experiments on chromosomes 1, 2 and 3 for GM12878 and K562 (Fig. 1). We found that the majority of CpG sites were either hyper-methylated or hypo-methylated. Specifically, for GM12878 on chromosomes 1, 2 and 3, 67.73% of CpG sites have methylation level <0.1 (hypo-methylated), and 14.40% of CpG sites have methylation level >0.9 (hyper-methylated). Similarly, for K562, 60.42% are hyper-methylated and 14.43% are hypo-methylated. Based on this analysis, in order to balance the number of samples in methylated and un-methylated classes for leave-one-out cross-validation and blind test, we set the threshold β to be 0.01 making about half (46.25%) of the samples labeled as un-methylated. The threshold α was set accordingly to ensure the number of methylated samples was equal to the one of un-methylated samples. In this way, most of the samples were labeled into one of the binary classes, and no sample was labeled twice.

### Optimizing stacked denoising autoencoders (Benchmark 1)

The parameters of stacked denoising autoencoders include number of hidden layers, number of hidden units in each layer, pre-training learning rate, number of pre-training epochs, fine-tuning learning rate, and the maximum of training epochs.

We optimized the parameters to obtain the best average performance on the individual test samples in every round of leave-one-out cross-validation (Table 1). In each round, one sample was chosen as the test sample, and the other samples were equally split into one training set and one validation set. The training set was also used for unsupervised pre-training of the SdAs. We found that after the SdA architecture reached 23-500-500-2 (23 input unites, two hidden layers each with 500 hidden units, and two output nodes), the performance no longer changed when increasing the number of hidden layers and number of nodes in each layer. Therefore, the number of hidden layers was set to two, and the number of hidden units in each layer was set to 500 for the leave-one-out cross-validation and blind test. The setup of other parameters of SdAs can be found in the Methods section.

### Leave-one-out cross-validations for support vector machines and stacked denoising autoencoders (Benchmark 1)

**Figure 2:**
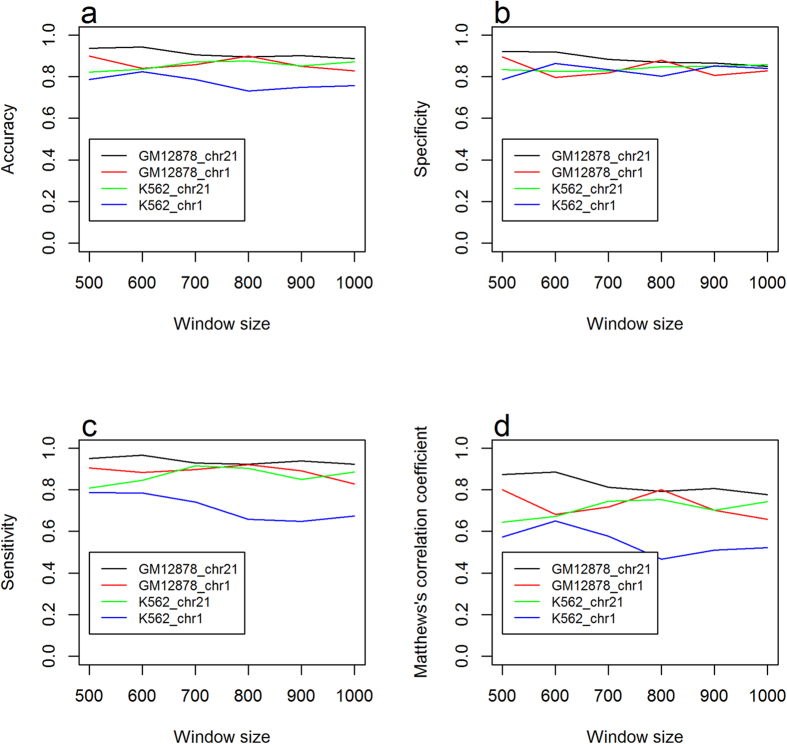
Leave-one-out cross-validation performance of SVMs with different window sizes, chromosomes, and cell lines. (**a**) prediction accuracy, (**b**) specificity, (**c**) sensitivity, and (**d**) Matthews’s correlation coefficient.

In order to compare the performance of SVMs, leave-one-out cross-validation was conducted on chromosomes 1 and 21 for GM12878 and K562 with different window-A sizes (Fig. 2). The output of the SVM classifier is a continuous number. Thus, we defined a cutoff μ to classify the output to discrete classes (for details see Methods).

By the comparison of accuracies (Fig. 2a) and Matthew’s correlation coefficient (Fig. 2d) of the two cell lines on chromosome 21 (black and green line) and chromosome 1 (red and blue line), we found that the performance for GM12878 is better overall than the performance for K562 on both chromosomes 1 and 21 on SVM model. One of the reasons may be that the number of samples for GM12878 is higher than K562 (Supplementary Table S1), resulting from the different Hi-C coverages in the two cell lines. The average number of Hi-C reads for each nucleotide on GM12878 (chromosome 1 with 1.415 and chromosome 21 with 0.822) is higher than K562 (chromosome 1 with 0.371 and chromosome 21 with 0.206). In Benchmark 1, we included all the window-Bs that have at least one Hi-C contact with window-A. A higher Hi-C coverage results in more samples having at least one Hi-C contact and a higher number of window-Bs on average for each target CpG site.

For both K562 and GM12878, SVMs achieve better performance on chromosome 21 than on chromosome 1 with most window sizes (Fig. 2a,d). However, the average specificity of prediction for K562 chromosome 1 does not have a significant difference with prediction on chromosome 21 (Fig. 2b). Together with the lower sensitivity on chromosome 1 (Fig. 2c), it indicates that the worse performance on chromosome 1 may be due to the worse performance on predicting true positive (methylated) samples.

**Figure 3:**
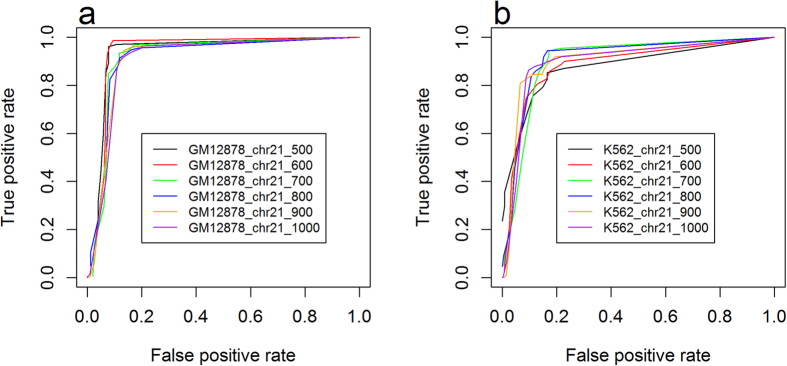
ROC curves of leave-one-out cross-validation using SVMs with different window sizes for (a) GM12878 and (b) K562.

The ROC curves were generated for chromosome 21. We calculated the values in the ROC curves by varying the cutoff μ from −2–2 (Fig. 3). There is not a common window size to obtain the best performance for all cell lines. Figure 3 suggests that 600 nt is the best window size for GM12878 chromosome 21, which achieves an accuracy of 94.3%, Matthews’s correlation coefficient of 0.886, specificity of 0.919 and sensitivity of 0.966 (based on Fig. 2). For K562, 800 nt is the best window size, which achieves an accuracy of 87.6%, Matthews’s correlation coefficient of 0.753, specificity of 0.848 and sensitivity of 0.904. Table 2 summarizes the best performance that SVMs achieve and the corresponding window sizes.

To compare the performance between SdAs and SVMs, we conducted leave-one-out cross-validations for an SdA on chromosomes 1 and 21 with window size 600nt (Table 3). On chromosome 21, SdA model obtained a worse performance for GM12878 with an accuracy of 0.935 compared to SVM’s accuracy 0.943. However, on chromosome 1, the SdA model achieved a better performance with an accuracy of 0.885, which is higher than SVM’s 0.839. Table 3 shows that the number of samples for chromosome 1 (2,616) is about six times higher than the ones for chromosome 21 (296), which may be one of the reasons for the performance difference. This indicates that the SdA algorithm may need more training samples to achieve better performance, whereas the SVM algorithm can achieve a decent performance with a much smaller size of training data.

### Evaluating SVMs and stacked denoising autoencoders on blind test data sets (Benchmark 1)

We further evaluated the performance of SVMs and SdAs using two blind test data sets. The predictive models were trained using CpG sites on chromosomes 1, 2 and 3 with different window sizes for both healthy (GM12878) and cancer (K562) cell lines. We used CpG sites on chromosomes 1, 2 and 3 as the training set because chromosomes 1, 2 and 3 are the largest three chromosomes in humans. The numbers of training samples associated with various window sizes are shown in Supplementary Figure S1. All features for the SVM and SdA classifiers were generated from the same dataset. The CpG sites on chromosomes 21 and X were selected as two independent test sets, considering that chromosome 21 is a smaller chromosome and chromosome X can be inactivated by the lncRNAs called Xist for female^42^. It would be interesting to study the methylation pattern in X chromosome and compare it with chromosome 21.

**Figure 4:**
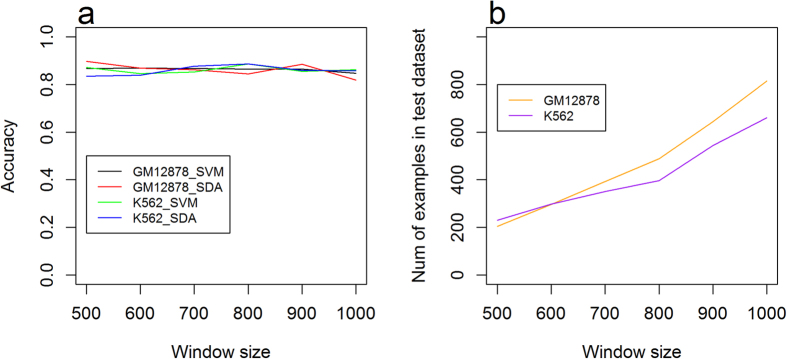
(**a**) Accuracy of blind test on chromosome 21 using SdAs and SVMs. (**b**) Number of samples in the test dataset with different window sizes in chromosome 21.

**Figure 5:**
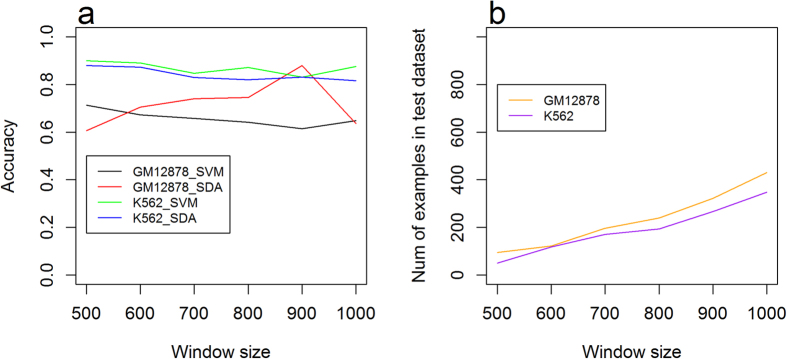
(**a**) Accuracy of blind test on chromosome X using SdAs and SVMs. (**b**) Number of samples in the test dataset with different window sizes in chromosome X.

Comparing to chromosome X, predictions on chromosome 21 for GM12878 achieved a better performance on most window sizes (Figs 4 and 5). The difference may be due to the chromosome-specific methylation patterns. We explored the distribution of the methylation level on chromosomes 21 and X (Supplementary Figs S2 and S3), which suggests that for both GM12878 and K562, methylation distributions on chromosome 21 share similar patterns with the distribution on chromosomes 1, 2, and 3 (Fig. 1), which were used as the training data. For example, on chromosome 21, 51.98% of the CpG sites have a methylation level <0.1, and 15.44% of the CpG sites have methylation level >0.9 (Supplementary Fig. S2), which is similar to 67.73% and 14.40% in chromosomes 1, 2, and 3 (Fig. 1), respectively. However, for GM12878 on chromosome X, CpG sites with methylation level <0.1 take a much lower proportion, that is, 33.24% (Supplementary Fig. S3), which indicates that the methylation distribution in chromosome X is significantly different from the distribution in chromosomes 1, 2, 3 (training data set), and 21 (the other test data set).

### Predicting methylation state of lncRNA loci (Benchmark 1)

We investigated DNA methylation prediction for CpGs sites located within lncRNAs genes. We used the same training data set, which is the combination of CpG sites on chromosomes 1, 2, 3, and the test data set that contains the CpG sites within lncRNAs genes on chromosomes 21 and X.

#### Benchmarking on chromosome 21 lncRNA loci

**Figure 6:**
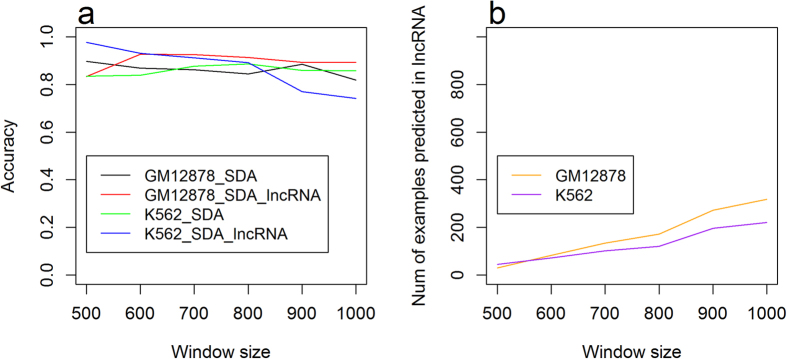
(**a**) Performance of SdAs for the prediction of methylation for lncRNAs and CpG sites without region-specific limitation on chromosome 21. (**b**) Number of samples in the test dataset on different window sizes in chromosome 21.

for both GM12878 and K562 on chromosome 21, predictions for CpG sites within lncRNAs (Fig. 6) achieved better performance than the ones without region-specific limitation (that is, both CpG sites within lncRNA genes and outsides lncRNA genes) (Fig. 4). Specifically, for K562, an SdA reached the best accuracy of 0.977, while the best accuracy is 0.886 for predictions on all CpG sites. This improvement in accuracy may be because the methylation distribution patterns of chromosome 21 lncRNAs are more similar to the training dataset (chromosomes 1, 2 and 3) as compared to the ones of all CpG sites. Thus, we explored the methylation patterns of CpG sites within lncRNA on chromosome 21 (Supplementary Fig. S4). We found that 60.42% of the CpG sites within lncRNAs had a methylation level <0.1 (Supplementary Fig. S4), which is closer to the training dataset’s 67.73% (Fig. 1) than 51.98% in CpG sites without region-specific limitation (Supplementary Fig. S2).

#### Benchmarking on chromosome X lncRNA loci

**Figure 7:**
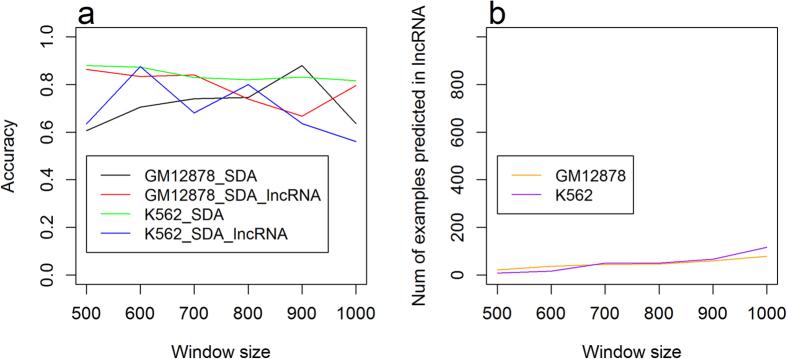
(**a**) Performance of SdAs for the prediction of methylation for lncRNAs and CpG sites without region-specific limitation on chromosome X. (**b**) Number of samples in the test dataset on different window sizes in chromosome X.

furthermore, we found that the performance for lncRNAs genes of GM12878 on chromosome X is worse than the one on chromosome 21 (Figs 7 and 6). The difference of performance for GM12878 on chromosome 21 and X may result from the different characteristics of methylation for chromosomes 21 and X. Therefore, we explored the distributions of the methylation levels of lncRNAs for chromosomes 21 and X (Supplementary Figs S4 and S5). It can be found that for GM12878, the methylation distribution of chromosome 21 lncRNAs shares similar patterns with the methylation levels of all CpG sites (not only lncRNAs) on chromosomes 1, 2, and 3, which were used as the training data. Specifically, on chromosome 21, there are 61.57% of lncRNA CpG sites having a methylation level <0.1 (Supplementary Fig. S4), which is similar to 67.73% on chromosomes 1, 2 and 3 (Fig. 1). In contrast, on chromosome X, only 31.65% of the lncRNA CpG sites have a methylation level <0.1 (Supplementary Fig. S5), which indicates that the methylation distribution for lncRNA on chromosome X is quite different from the training data set comprised of chromosomes 1, 2 and 3.

Moreover, since both K562 and GM12878 are samples from female, it is possible that the X chromosome may be inactivated or in the process of inactivation by an lncRNA called Xist^42^ that packs the three-dimensional structure of X chromosome to disable the expressions of most X-chromosome genes. The change of three-dimensional genome structure of X chromosome influences the genome structural based features used in our methods and may also alter the DNA methylation patterns in chromosome X. Moreover, another reason may be that the test dataset of X chromosome is relatively small, compared to the one of chromosome 21. Therefore, the influence of error becomes more significant (Fig. 7b).

### The impact of Hi-C based genome topological features (Benchmark 2)

Benchmark 1 used the methylation level of sequential neighboring region of a target CpG site. In Benchmark 2, we eliminated that feature in order to benchmark the performance only based on the sequence composition of window-A and window-B (three-dimensional topological neighboring regions) in addition to methylation levels in window-B. Compared to Benchmark 1, we added 74 PseTNC features for window-A and eight features for window B. All of these newly added features indicate sequence composition. In Benchmark 2, we used both up-sampling and down-sampling to balance training data. Details can be found in the Methods section.

We also changed Hi-C based window-B to a randomly generated window-B in order to observe the impact of Hi-C inferred topological neighbors. We only used the randomly generated windows that do not have any Hi-C contact with the Hi-C ranges (the region surrounding a target CpG site that Hi-C neighbors were collected from, see the Methods section for details). In this way, we eliminated topological neighbors from the random windows. Different sizes of Hi-C ranges were tested.

Table 4 shows the 5-fold cross-validation accuracy and MCC scores of SVMs on using both Hi-C based window-Bs and randomly selected windows. The performance on Hi-C based and random windows is similar in this case with random windows performing slightly worse. Table 5 and Supplementary Tables S2–S6 show the performance of SdAs. We benchmarked one, two, and three hidden layer(s) and found that more hidden layers result in significantly worse performance for randomly selected windows.

In order to benchmark the influence of unsupervised pre-training of SdAs, we conducted three independent 5-fold cross-validations, in which the epochs of unsupervised pre-training and supervised training were set to (10, 100) (Table 5 and Supplementary Table S2), (10, 10) (Supplementary Tables S3 and S4), and (100, 10) (Supplementary Table S5 and S6) while unifying all the other factors including pre-training, training, validation, and testing data and other SdA parameters. The results show that larger epochs for unsupervised pre-training and smaller epochs of supervised training may decrease performance and make the SdAs perform significantly worse for random windows. The epochs of unsupervised pre-training and supervised training of (10, 100) generated the best performance.

### Blind test on chromosome 21 and lncRNA loci (Benchmark 2)

We tested the performance on randomly combined samples from chromosomes 1 and 21 (details of data generation see Methods). For SdAs, the epochs of unsupervised pre-training and supervised training was set to (10, 100), and the other parameters remained the same as the 5-fold cross-validation that generated the best results. Similar findings as in the 5-fold cross-validation were observed. That is, a higher number of hidden layers makes the SdAs perform significantly worse on the random windows in GM12878 (Table 6). For K562, the SdA model achieved an accuracy of 72.01%. We also benchmarked the performance on the CpG sites without genome topological features (no Hi-C signals); and in this case the accuracies of GM12878 and K562 are 84.25% and 69.95% respectively.

Using the optimized SdA configuration and SVM model found in the 5-fold cross-validations, we tested their performance on GM12878 chromosome 21 lncRNA loci (Table 7). Two hidden layers of SdAs generated the best testing accuracy that is similar to SVMs. In terms of MCC score, a two-hidden-layer SdA (0.6427) performed slightly better than an SVM (0.6385).

### Benchmarking the parallel algorithm for generating features and training SVMs

A parallel algorithm was used to reduce the execution time of the entire feature generation process. The parallel algorithm was implemented using C++ and MPICH, and the performance tests were conducted on our own shared memory server equipped with 48 CPUs with speed 1200 MHz and 126 gigabytes of memory. A test result is given in Supplementary Table S7, which shows that our parallel method dramatically saves computational time.

## Discussions

We developed SVM and SdA models to predict binary methylation state of CpG sites on GM12878 and K562 on different chromosomes with different window sizes. In the leave-one-out cross-validation for SVM classifier, the accuracy reaches 0.943 on chromosome 21 of GM12878, while the accuracy reaches 0.876 on chromosome 21 of K562. The distinction of performance between GM12878 and K562 on the SVM model may result from the different numbers of samples and Hi-C coverage. This indicates that the Hi-C reads coverage plays an important role, as a higher Hi-C coverage can increase the resolution of the three-dimensional genome structure and provide more neighboring CpG sites as features for the machine learning models.

Furthermore, we evaluated the SdA classifier using a leave-one-out cross-validation. For SdA classifier tested on 296 CpG sites of GM12878 chromosome 21, the accuracy reaches 0.935, which is slightly lower than SVM classifier’s 0.943. However, on chromosome 1 for GM12878, in which the total number of leave-one-out samples reaches 6,516, the accuracy of SdA classifier reaches 0.885, which is obviously higher than SVM classifier’s 0.839. The difference of performance between SVMs and SdAs may suggest that the SdA algorithm needs more training samples to achieve better performance. Moreover, by comparing the performance with features excluding methylation level of neighbors and GC contents, we found that, especially for SdAs, neighboring methylation levels and GC content are influential to the prediction performance.

Moreover, we evaluated the performance of SVM and SdA classifiers using two blind test sets. Our experiments used chromosomes 1, 2 and 3 as the training set, and chromosomes 21 and X as two independent test data sets. An SdA reaches the best accuracy of 0.897 on chromosome 21 of GM12878 with window size 500nt. On chromosome 21, both SVMs and SdAs have a stable performance over different window sizes for both K562 and GM12878. For chromosome X, an SdA achieved a best accuracy of 0.880 for GM12878 with window size 900 nt. Overall, the accuracies of GM12878 on chromosome X are lower than the ones on chromosome 21 for most window sizes. This may be because the distributions on chromosome X are largely different from the distributions in the training dataset of chromosomes 1, 2 and 3.

We investigated the performance of predicting the DNA methylation state for CpG sites within lncRNA DNA locus. The best accuracy, 0.977, was obtained when using an SdA on chromosome 21 of K562 with window size 500nt. We further found that the performance on chromosome X was overall worse than the performance on chromosome 21. By analysis, we found the methylation distribution of lncRNA genes in chromosome X of GM12878 was largely different from the distributions found in both chromosome 21 and the training chromosomes 1, 2 and 3. This may result from the existence of an lncRNA called Xist that packs and inactivates the chromosome X of female causing the different methylation patterns. Our data indicates methylation patterns of lncRNA may be chromosome- and cell line-specific.

In order to benchmark the influence of Hi-C based genome topological features, we replaced Hi-C neighbors with randomly selected windows and then benchmarked the performance. We found that using random windows significantly decreased the performance of SdAs with two or more hidden layers. We also tested it with different numbers of epochs for pre-training and fine-tuning and found that a larger number of fine-tuning increases performance whereas a larger number of pre-training decreases the performance.

## Methods

### Datasets

#### Human cell lines

The cell lines GM12878 and K562 were selected for our study because of their accessibility and sufficient experimental data associated with them. GM12878 is a B-lymphocyte cell line from a female, while K562 is an immortalised cell line from a female patient with chronic myelogenous leukemia (CML) (for description of these two cell lines see http://www.genome.gov/26524238). Thus, investigating the methylation prediction on these two cell lines may help us characterize the methylation patterns of cancer and healthy cell lines.

#### DNA methylation data

DNA methylation state at each CpG dinucleotide is measured by Reduced Representation Bisulfite Sequencing (RRBS) data. RRBS methylation data for cell lines GM12878 and K562 were obtained from the ENCODE project (http://hgdownload.cse.ucsc.edu/goldenPath/hg19/encodeDCC/wgEncodeHaibMethylRrbs/).

#### Genome topology

The Hi-C paired reads^36^ for GM12878 and K562 cell lines were obtained from the public accessible NCBI GEO database (http://www.ncbi.nlm.nih.gov/geo/query/acc.cgi?acc=GSM1181867) and NCBI SRA database (accessible at http://sra.dnanexus.com/experiments/SRX011614/runs), respectively. The paired-end Hi-C reads were mapped to the human reference genome (UCSC version hg19) using the read sequence alignment tool Maq^43^. The contact library containing spatial contacts between pairs of genomic positions were generated by parsing the Maq mapping outputs. Each contact between two positions on genome implies that they are spatially proximate in three-dimensional structure.

### Support vector machines (SVMs)

There are four types of kernel functions in SVM-Light^44^: linear, polynomial, radial basis function, and sigmoid. In Benchmark 1, we selected the polynomial kernel function for our SVM classification model because this kernel function achieves the best performance based on the 23 features using leave-one-out cross-validation (data not shown). Based on the optimization of SVM model, the parameter C (trade-off between training error and margin) was set to 5, and the polynomial kernel function parameter d was set to 3. In Benchmark 2, the radical basis function was selected as the kernel function based on the cross-validations on 109 features. We used the default value of parameter C in SVM-light and set the parameter gamma in radical basis function to 9 based on optimization.

### Deep learning - Stacked Denoising Autoencoder

The deep learning architecture applied to this research is Stacked Denoising Autoencoder (SdA)^45^ implemented with Theano (http://deeplearning.net/software/theano/). Theano is a Python-based library enabled GPU-based high performance computing for deep networks. The SdA algorithm composed of two phases of learning. The first phase is unsupervised pre-training carried out by layers of denoising autoencoders, which learn a reconstruction *Z* from corrupted version of data *X* by minimizing the cross-entropy of the reconstruction:


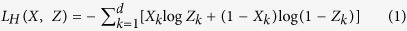


for all the training samples in a minibatch. The *Z*, which is the reconstruction of the corrupted version of data *X*, was computed from


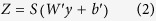


where 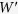 is the reconstruction weighting matrix, 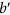 is the reconstruction bias, and function *S*() is a sigmoid function:


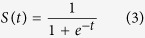


Also, Z, the reconstruction of the corrupted input *X*, can be considered as the prediction of *X* because it tries to have the same shape of *X* given *y*, where


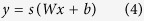


in which *s*() is a sigmoid function, *W* is the weighting matrix, and *b* is the bias. Formula 4 maps the corrupted input 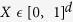 to a hidden representation 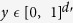, which is reversely mapped by Formula (2) to build a reconstruction of corrupted data X by minimizing Formula (1). The corrupted input *X* is a sparse version of the original input *X-orig*. There are multiple ways to generate *X* from *X-orig*, and we used a parameter called corruption level to set it. The hidden units in a hidden layer were randomly selected to be disabled from an input node based on the probability set by the corruption level parameter. This corrupted version of autoencoders does not only learn the identifies of the input data, but also learns the features that are more useful to the problem. Therefore, it is also named denoising autoencoders^46^. This corruption process was applied in each layer of hidden units in stacked denoising autoencoders.

The learning process computes the cost based on Formula (1) for each layer of stacked denoising autoencoders and updates the weights and biases by gradient descent. The training process starts from the first layer directly connecting to the input data, and continues layer by layer. The trained *m* layers enable the computation of latent representation in layer *m* + 1. In this way, all stacked layers of denoising autoencoders were trained, and the outputs from these layers of denoising autoencoders are the reconstruction of input *X-orig* or the features selected from the original data. With that, the unsupervised pre-tuning part is finished.

A supervised fine-tuning is applied after the unsupervised pre-tuning. A logistic regression model was added on top of the layers of denoising autoencoders that calculates:


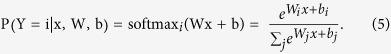


This formula calculates the probability of an input vector *x* having the class *i* of *Y*. W is the weighting matrix; b is the bias; and j can be all the possible classes in *Y*. After calculating the probabilities for all possible classes in *Y*, an input vector x is assigned or predicted to the class that gives highest probability as:


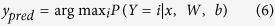


A multilayer perceptron is constructed that shares the same number of layers, number of neurons in each layer, weight, and bias as previously trained stacked denoising autoencoders. Label value *Y* was used to train the multilayer perceptron by a backpropagation algorithm with logistic function as activation function. In this way, the multilayer perceptron was trained, and the entire learning architecture was fine-tuned. Therefore, the weights and bias in each hidden layer of the deep network were updated again based on the class label *y* of each training sample.

Based on our Benchmark 1 (see **Results**), the best configuration achieving optimal performance contains two hidden layers each with 500 hidden units; the pre-training learning rate and epochs were set as 0.01 and 100; the fine-tuning learning rate was set as 0.1; and the maximum of training epochs was set as 1000. In Benchmark 2, the learning rates of pre-training and fine-tuning were set to 0.01; corruption level was set to 0.1 for all hidden layers. Different epochs for pre-training and fine-tuning were tested (see Results). The SdA algorithm was implemented on a NVIDIA Quadro K5100 GPU with 1,536 CUDA parallel processor cores.

### Machine learning features

#### Overview

we defined two types of windows for each CpG site to generate features. The first type of window, window-A, is a DNA sequence window with the target CpG site as the center whose size varies from 500–1000 nt. Window-A was used to generate features from the sequences that are immediately adjacent to the target CpG sites. The second type of window, window-B, is a sequence window with point X in the center, whereas point X and a point in window-A (for Benchmark 1) or “Hi-C range” (for Benchmark 2, definition see below) must be in contact indicated by a Hi-C paired ends read. The coordinates of CpG sites and corresponding window sequences were determined based on human reference genome hg19.

Features from window-A: there are four types of DNA nucleotides: adenine (A), thymine (T), guanine (G), and cytosine (C). Both the ratio and order of these four nucleotides indicate important features of the DNA sequence. Studies^27^,^33^ have proved that the occurrence of certain DNA patterns may be related to the methylation level. Hence, for Benchmark 1, the ratios of A, T, G, C and eight specific fragments (sequential signatures, Table 8), which have been proven to be useful features for methylation prediction^25^, were used as features for our prediction. In some recent studies^30^, the methylation state of neighboring regions was incorporated as one of the features. Hence, the “percentMeth” values from RRBS experiments indicating averaged methylated percentage were gathered and averaged in window-A and then were included as a type of feature in Benchmark 1.

For Benchmark 2, that is, the 5-fold cross-validation on chromosome 1 and blind test on random combination of chromosomes 1 and 21, we incorporated more sequential features and eliminated the features indicating methylation level in neighboring region. As introduced by some recent publications^47^,^48^,^49^,^50^,^51^,^52^,^53^,^54^,^55^,^56^,^57^,^58^,^59^,^60^,^61^,^62^, some useful statistical features for biological systems have been developed and presented. These features include pseudo amino acid composition (PseAAC)^63^, pseudo k-tuple nucleotide composition (PseKNC) and pseudo trinucleotide composition (PseTNC)^64^. We implemented 74 PseTNC features as DNA sequence property features. The pseTNC (pseudo trinucleotide composition) is a statistical feature, which incorporates the occurrence frequencies of all the pseudo trinucleotide compositions. The features are defined as


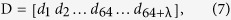


in which the first 64 features measure the local or short-range sequence pattern and the next λ = 10 components measure the global effect. The 74 features were generated by incorporating the frequency and multiple physical properties of each pseudo trinucleotide composition. The detail of calculating these features can be found in the ref. 64.

Features based on three-dimensional genome topology - Benchmark 1: for each target CpG site, we gathered all the Hi-C contact pairs with one end falling into the window-A region. Using the other Hi-C end as the center, a window-B was defined with the same size of window-A. We only included the window-Bs that are >1000 nt away from the target CpG sites ensuring they are sequentially a long-distance away but proximate in three-dimensional space. In this way, we eliminated the methylation level of the sequentially neighboring region for a target CpG site. Because multiple Hi-C pairs may have one end falling into the window-A region of each target CpG site, we usually gathered multiple window-Bs. The number of available window-Bs is influenced by the size of window-A and the Hi-C reads coverage, which was calculated by: multiplying the length of Hi-C read by the number of Hi-C reads and then dividing by the total length of the reference genome. We benchmarked our performance with different sizes of window-A. For each window-B, we generated the DNA sequence properties (Table 8) and averaged methylation PercentMeth, and then averaged these values for multiple window-Bs.

Benchmark 2: we eliminated the methylation level in window-A, but only kept the methylation levels in window-B for every target CpG sites. In this way, the prediction models no longer know the methylation level in the sequential neighboring region of a target CpG site, increasing the prediction difficulty. In order to observe how the number of Hi-C neighboring regions impact prediction performance, a “Hi-C range” was defined with the target CpG site as the center of it. The Hi-C pairs with one end fell into this “Hi-C range” and were collected; and the other end was used as the center of window-B. Only the Hi-C contacts whose two ends have a sequential distance longer than the “Hi-C range” were included so that only long-range spatial neighbors were kept.

### Evaluation methods

#### Evaluation criteria

the specificity (Sp), sensitivity (Se), accuracy (Acc), and Matthews’s correlation coefficient (MCC) were used to evaluate prediction performance. These parameters were calculated using the following equations^65^:


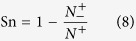



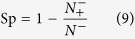



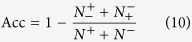



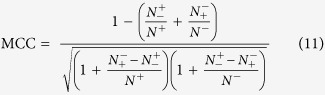


where 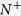 is the total number of the positive samples (methylated samples), and 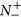 is the number of the positive samples incorrectly predicted as negative samples (un-methylated samples), 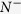 is the total number of the negative samples, and 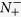 is the number of negative samples incorrectly predicted as the positive samples.

Receiver Operating Characteristic (ROC) curves for leave-one-out cross-validation were plotted with different values of threshold μ, which was used as the cutoff for methylated and unmethylated classes based on the SVM output real-number value.

### Leave-one-out cross-validation (Benchmark 1)

The performance of SVM model and SdAs were evaluated by the leave-one-out cross-validation. For the prediction of methylation state of each CpG site, the rest of the CpG sites were used as training samples. For SdAs, the rest of the samples were split so that 50% of the samples were used as fine-tuning set, and 50% as validation set. The same fine-tuning samples were also used in the pre-training stage (unsupervised learning), in which the target values Y were not used. The final evaluation of the prediction performance was obtained by averaging the results from all rounds of cross-validation.

The methylation value for each CpG site was indicated by the value of percentMeth from RRBS experiments. Methylation level of a CpG site is a continuous value ranging from 0 (un-methylated) to 1 (methylated). Because we tried to classify the methylation status of a CpG site into binary classes (methylation state), that is, either methylated or un-methylated, we incorporated two thresholds α and β to convert the continuous value of PercentMeth into binary classes. Specifically, if the PercentMeth value of a CpG site is larger than α, the CpG site is classified as methylated, and if the methylation level of a CpG site is less than β, the CpG site is classified as un-methylated (methylation-resistant). The threshold β was set first to 0.01, and then the threshold α was calculated based on β to ensure these two binary classes would have equal numbers of samples. Balancing the number of samples in each class avoids bias in training.

### Blind test on chromosomes 21 and X (Benchmark 1)

The chromosomes 1, 2 and 3 were used as training data sets because of their relatively larger size, and chromosomes 21 and X were selected as two independent blind testing data sets because of their smaller size and the possible inactivation of female X chromosome.

### Five-fold cross-validation (Benchmark 2)

In Benchmark 2, we eliminated the feature “Ave_meth” (methylation level in the neighboring region of target CpG sites) and added 74 PseTNC features (Table 8). We collected all the CpG sites with “PercentMeth” value in the RRBS experiment equal to 0 and assigned them as un-methylated samples; the CpG sites with >=0.9 were used as positive samples. In this way, we collected in total 559 positive samples and 1,959 negative samples. These samples were evenly split into five folds. For the training of SVMs, down-sampling (cut samples from the majority class) was performed on the four training folds. The up-sampling technique (randomly picking up the same number of samples for the minority class) was performed for SdAs in order to balance the positive and negative samples in the training folds. The data in the testing fold was not balanced. For SdAs, three folds were used as fine-tuning data (up-sampling balanced), one fold as validation (up-sampling balanced), and one fold as test (not balanced). Benchmark 2 was performed on chromosome 1 of the GM12878 cell line.

In order to benchmark the contribution of unsupervised pre-training of SdAs, we randomly collected 2,330 samples on chromosome 1 with unknown target value. For every round in the 5-fold cross-validation and blind test with chromosome 21, this data set was used as the pre-training sample for training SdAs. This 5-fold cross-validation was performed with multiple “Hi-C ranges” (definition see Machine Learning Features section).

### Blind test with Chromosome 21 (Benchmark 2)

We collected 1,039 positive and 1,746 negative samples from chromosome 21 in the same way as from chromosome 1, and randomly combined them with all the samples from chromosome 1 used in the 5-fold cross-validation. All of the randomly combined data set was split into five folds. For SVMs, four folds were used to train the model and one for test. For SdAs, three folds were used as fine-tuning, one fold for validation, and one for testing. The same un-labeled data set was used for unsupervised pre-training. No up-sampling or down-sampling was performed on any of the folds. Only 10K “Hi-C range” was used in this blind test stage.

### Test with randomly selected windows (Benchmark 2)

To benchmark the contributions of Hi-C related features, we replaced Hi-C based window-Bs with same-size random windows, which do not have any Hi-C contacts with the “Hi-C range” of a target CpG site. All the same features were generated on the random window as for Hi-C window-B.

### Parallelization of feature generation and SVM classification

A parallel algorithm was designed to reduce the execution time of feature generating and SVM-light classification. First, multiple processors simultaneously read the Hi-C contact files using MPI (Message Passing Interface), and then a parallel version of SVM-light was developed to make each processor perform learning and classification simultaneously. This parallel algorithm was designed and tested on an early version of our methods that targeted on predicting average methylation level of a segment of the genome instead of each CpG site. However, the feature types and SVM classification are the same. Execution time decreased with the increase of number of processors (see the Results section).

### Statement for experiments involving vertebrates and human subjects

This research was conducted with purely computational methods and did not use any animals, human subjects, or tissue samples. This work did not conduct any wet lab biological experiments that used vertebrates, human subjects, or tissue samples. The data of all cell-lines were downloaded from the public database ENCODE that has already been previously published.

## Additional Information

**How to cite this article**: Wang, Y. *et al.* Predicting DNA Methylation State of CpG Dinucleotide Using Genome Topological Features and Deep Networks. *Sci. Rep.* **6**, 19598; doi: 10.1038/srep19598 (2016).

## Supplementary Material

Supplementary Information

## Figures and Tables

**Table 1:** Performance of SdAs for GM12878 on chromosome 21 under different numbers of hidden layers and different numbers of hidden units using leave-one-out cross-validation.

Number of hidden units and hidden layers	200	200–200	500	500–500	500–500–500
Accuracy	0.889	0.891	0.896	0.935	0.935

**Table 2:** The best performance achieved from leave-one-out cross-validation using SVMs on chromosomes 1 and 21 for cell lines GM12878 and K562.

Cell Line	Chromosome	α	Window Size	Acc	Sp	Se	MCC
GM12878	CHR1	0.55	500	0.900	0.894	0.905	0.800
GM12878	CHR21	0.99	600	0.942	0.918	0.966	0.886
K562	CHR1	0.07	600	0.823	0.863	0.784	0.649
K562	CHR21	0.43	800	0.876	0.848	0.904	0.753

The threshold α was used to ensure equal number of samples in methylated class and un-methylated class.

**Table 3:** Performance of leave-one-out cross-validation using SVMs and SdAs on chromosomes 1 and 21 for cell line GM12878 on the window size 600 nt.

Classifier	Cell Line	Chromosome	Acc	Number of Samples
SdA	GM12878	CHR21	0.934	296
SVM	GM12878	CHR21	0.942	296
SdA	GM12878	CHR1	0.885	2616
SVM	GM12878	CHR1	0.839	2616

**Table 4:** The SVM’s 5-fold cross-validation accuracy and MCC scores of using Hi-C based topological neighboring window-Bs and random window-Bs on chromosome 1 with different Hi-C ranges.

Hi-C range	Acc (Hi-C based)	Acc (random)	MCC (Hi-C based)	MCC random
10K	0.831	0.828	0.616	0.600
20K	0.833	0.810	0.618	0.584
30K	0.830	0.815	0.614	0.586
40K	0.837	0.832	0.623	0.606
50K	0.838	0.824	0.628	0.601

**Table 5:** The 5-fold cross-validation accuracies of SdAs on chromosome 1 with different Hi-C ranges.

Hi-C range	Hi-C_1L	Random_1L	Hi-C_2L	Random_2L	Hi-C_3L	Random_3L
10K	0.829	0.830	0.837	0.714	0.835	0.406
20K	0.839	0.839	0.828	0.668	0.829	0.376
30K	0.840	0.835	0.832	0.823	0.830	0.376
40K	0.828	0.835	0.831	0.831	0.828	0.565
50K	0.841	0.819	0.826	0.828	0.834	0.326

The SdA model was trained with 10 pre-training epochs (unsupervised learning, learning rate 0.01) and 100 fine-tuning epochs (supervised learning, learning rate 0.01). The 1L, 2L and 3L are the number of hidden layers with corruption levels of all layers set to 0.1. All the layers have 100 hidden nodes. Features based on genome topological neighbors (window-Bs, indicated as “Hi-C” in the table) and features based on randomly selected regions (random windows, indicated as “Random” in the table) were used to benchmark the impact of Hi-C based features.

**Table 6:** The blind test accuracy and MCC scores for SdAs and SVMs on randomly combined training and testing samples from chromosomes 1 and 21 with Hi-C range 10 K.

Classifier	Features	SdA architecture	Acc	MCC
SdA	Hi-C based window-B	109-100-2	0.871	0.666
SdA	Random window-B	109-100-2	0.810	0.612
SdA	Hi-C based window-B	109-100-100-2	0.867	0.659
SdA	Random window-B	109-100-100-2	0.631	0.058
SVM	Hi-C based window-B	NA	0.860	0.685
SVM	Random window-B	NA	0.858	0.725

The ration of fine-tuning, validation, and testing samples for SdAs is 3:1:1. With two hidden layers, the MCC score of an SdA is 0.058. We found that the predictions are highly biased to negative samples. This causes the false negative to be a value close to 1. Therefore, it has a very low MCC score.

**Table 7:** Performance of SdAs and SVMs for predicting methylation level of CpG sites within lncRNA regions.

Classifier	SdA architecture	Acc	MCC	Number of test samples
SdA	109-100-2	0.796	0.5678	2138 (551 positive, 1587 negative)
SdA	109-100-100-2	0.784	0.5617	2138
SdA	109-100-100-100-2	0.832	0.6427	2138
SVM	NA	0.837	0.6385	2138

The SdA architecture and SVM model used were the one with the best test accuracy in 5-fold cross-validation on chromosome 1 (Table 5, Supplementary Tables S2–S6). The number of testing lncRNA samples are 2,138 (551 positive and 1587 negative).

**Table 8:** Features used for machine learning algorithms and their descriptions.

Feature name	Feature description	Used in benchmark:
Ra_A	Ratio of adenine in window-A	1, 2
Ra_B	Ratio of thymine in window-A	1, 2
Ra_C	Ratio of guanine in window-A	1, 2
Ra_D	Ratio of cytosine in window-A	1, 2
Pa_AAWGGR	Pattern frequency of AAWGGR in window-A	1, 2
Pa_TGRAAT	Pattern frequency of TGRAAT in window-A	1, 2
Pa_AAT	Pattern frequency of AAT in window-A	1, 2
Pa_ATGVAA	Pattern frequency of ATGVAA in window-A	1, 2
Pa_ACG	Pattern frequency of ACG in window-A	1, 2
Pa_GC	Pattern frequency of GC in window-A	1, 2
Pa_CG	Pattern frequency of CG in window-A	1, 2
Pa_TG	Pattern frequency of TG in window-A	1, 2
Pa_CCGC	Pattern frequency of CCGC in window-A	2
Pa_CCCC	Pattern frequency of CCCC in window-A	2
Pa_CGCC	Pattern frequency of CGCC in window-A	2
Pa_AAAG	Pattern frequency of AAAG in window-A	2
Pa_CTCC	Pattern frequency of CTCC in window-A	2
Ave_ meth	Average methylation level in window-A	1
PseTNC	74 pseudo tri-nucleotide composition features (Detail see Methods)	2
Ave_meth_Hi_C	Average methylation level in window-Bs	1, 2
Ave_Ra_A_Hi_C	Average Ra_A in window-Bs	1, 2
Ave_Ra_B_Hi_C	Average Ra_B in window-Bs	1, 2
Ave_Ra_C_Hi_C	Average Ra_C in window-Bs	1, 2
Ave_Ra_D_Hi_C	Average Ra_D in window-Bs	1, 2
Ave_Pa_AAWGGR_Hi_C	Average Pa_ AAWGGR in window-Bs	1, 2
Ave_Pa_TGRAAT_Hi_C	Average Pa_ TGRAAT in window-Bs	1, 2
Ave_Pa_AAT_Hi_C	Average Pa_ AAT in window-Bs	1, 2
Ave_Pa_ATGVAA_Hi_C	Average Pa_ ATGVAA in window-Bs	1, 2
Ave_Pa_ACG_Hi_C	Average Pa_ ACG in in window-Bs	1, 2
Ave_Pa_CCGC _Hi_C	Average Pa_CCGC in window-Bs	2
Ave_Pa_CCCC _Hi_C	Average Pa_CCCC in window-Bs	2
Ave_Pa_CGCC _Hi_C	Average Pa_CGCC in window-Bs	2
Ave_Pa_AAAG _Hi_C	Average Pa_AAAG in window-Bs	2
Ave_Pa_CTCC _Hi_C	Average Pa_CTCC in window-Bs	2
Ave_Pa_GC _Hi_C	Average Pa_GC in window-Bs	2
Ave_Pa_CG _Hi_C	Average Pa_CG in window-Bs	2
Ave_Pa_TG _Hi_C	Average Pa_TG in window-Bs	2

The feature names containing “Hi_C” were generated in window-B, that is, the topological neighbors indicated by Hi-C experiments.
