# Supplementary material for: Predicting DNA Methylation State of CpG Dinucleotide Using Genome Topological Features and Deep Networks
Source: Sci Rep. 2016 Jan 22;6:19598. doi: 10.1038/srep19598 (PMC4726425; doi:10.1038/srep19598)
Supplement: Supplementary Information [file srep19598-s1.pdf]

Supplementary materials for

Predicting DNA Methylation State of CpG Dinucleotides Using Genome Topological Features and Deep Networks

Yiheng Wang[1], Tong Liu[1], Dong Xu[2], Huidong Shi[3], Chaoyang Zhang[1], Yin-Yuan Mo[4], Zheng Wang*, [1]

* Corresponding author: Zheng Wang, zheng.wang@usm.edu

1. School of Computing, University of Southern Mississippi, 118 College Drive #5106, Hattiesburg, MS 39406, USA

2. Department of Computer Science and Christopher S. Bond Life Sciences Center, University of Missouri, 201 Engineering Building West, Columbia, MO 65211, USA

3. Department of Biochemistry and Molecular Biology, Georgia Regents University, 1120 15th Street, Augusta, GA 30912, USA

4. Department of Pharmacology and Toxicology, University of Mississippi Medical Center, 2500 North State Street, Jackson, MS 39216, USA

Author Emails:

YW: yiheng.wang@eagles.usm.edu

TL: tong.liu@eagles.usm.edu

DX: xudong@missouri.edu

HS: hshi@gru.edu

CZ: chaoyang.zhang@usm.edu

YM: ymo@umc.edu

ZW: zheng.wang@usm.edu

Supplementary Figures

Supplementary Figure S1 - Number of training samples generated from chromosomes 1, 2 and 3.

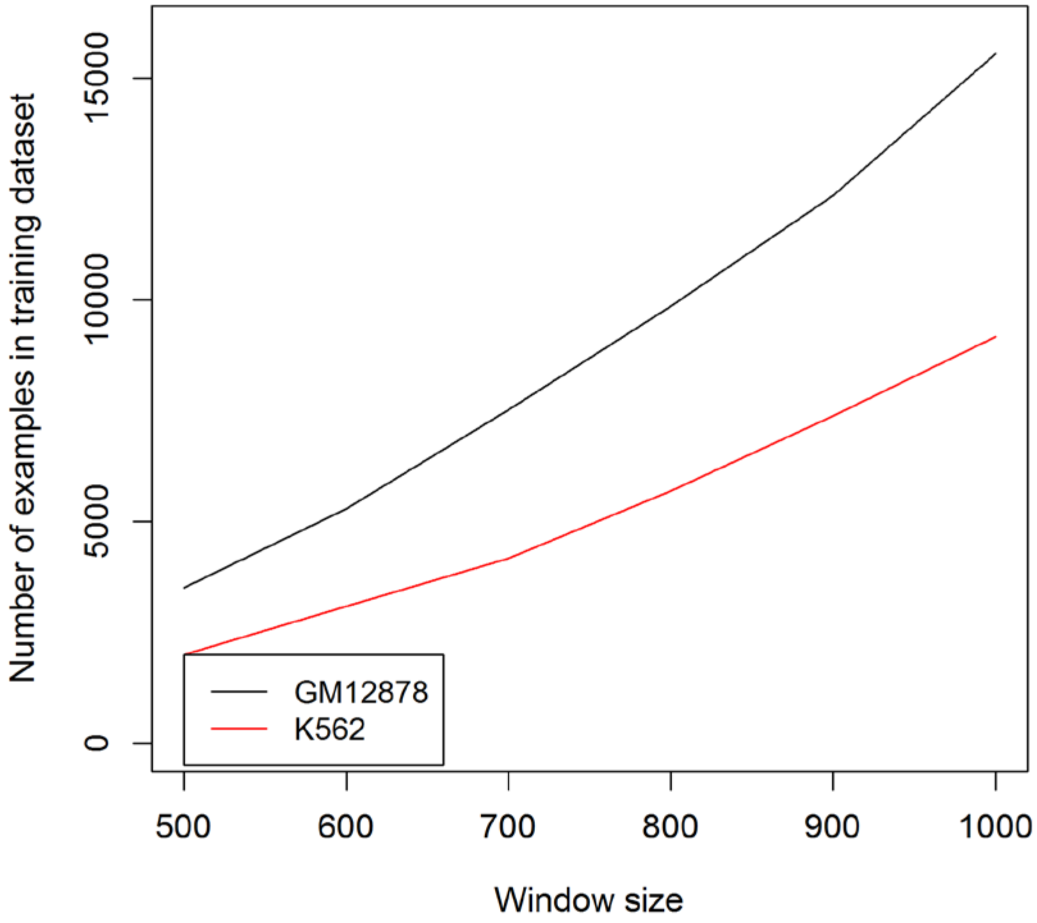

Supplementary Figure S2 - DNA methylation level distribution on chromosome 21 for GM12878 and K562.

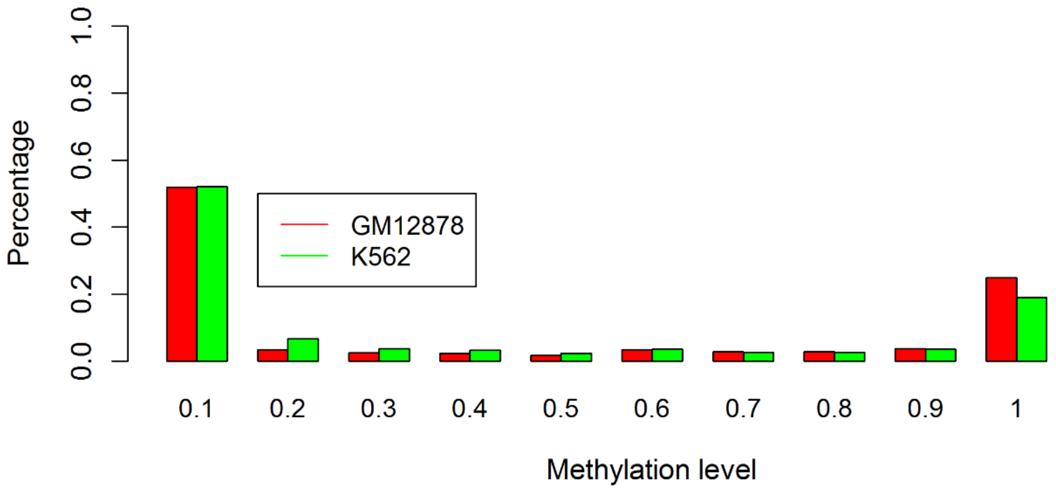

Supplementary Figure S3 - DNA methylation level distribution on chromosome X for GM12878 and K562.

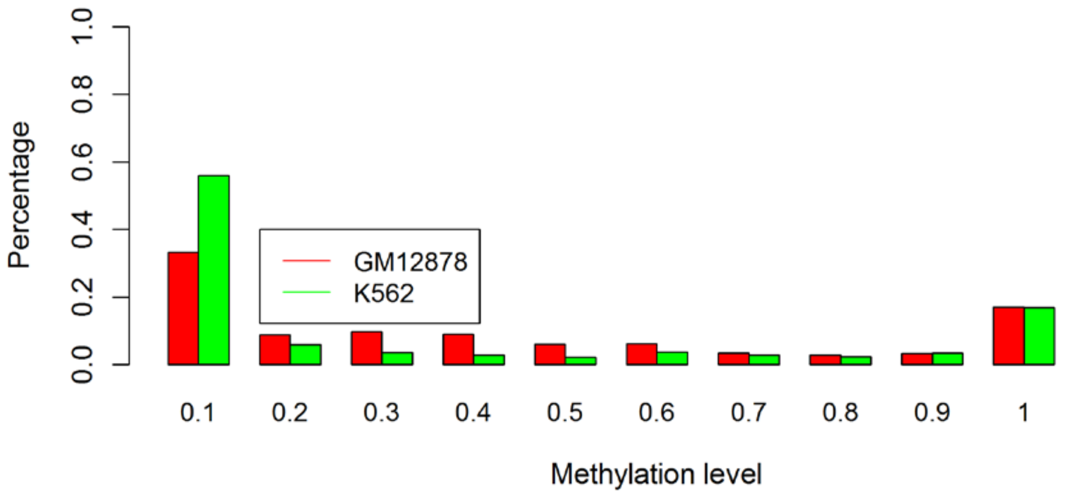

Supplementary Figure S4 - DNA methylation level distribution of CpG sites within lncRNA on chromosome 21 for GM12878 and K562.

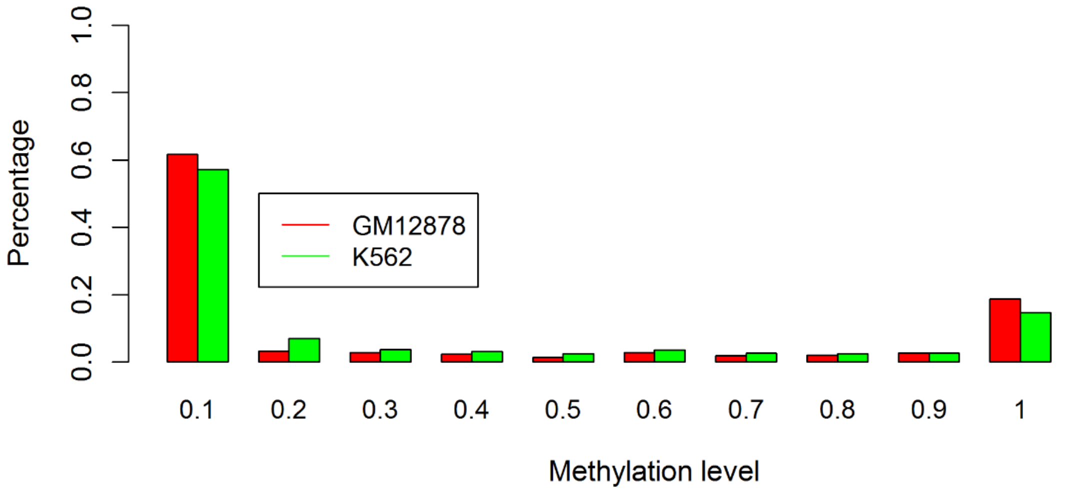

Supplementary Figure S5 - DNA methylation level distribution of CpG sites within lncRNA on chromosome X for GM12878 and K562.

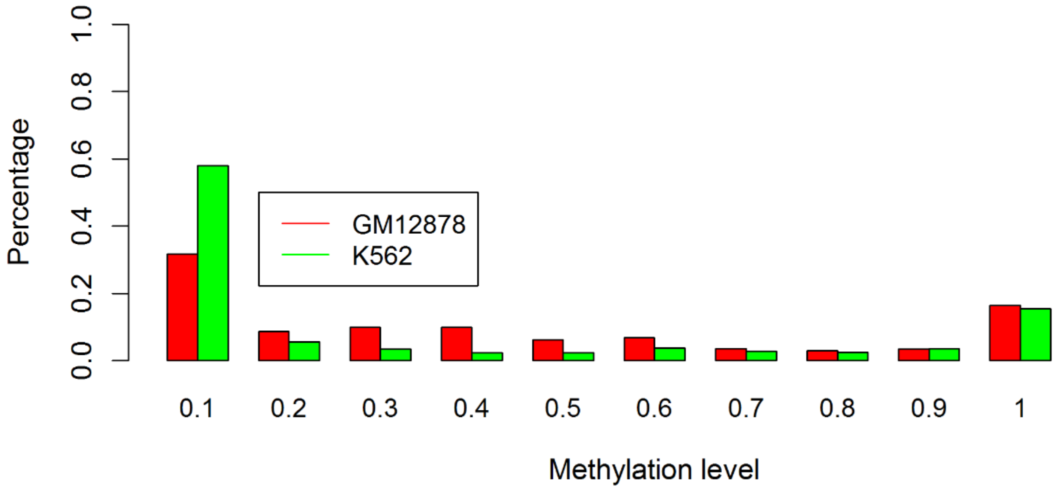

Supplementary Tables

**Cell Line**	**Chromosome**	**Number of Samples**
GM12878	21	296
K562	21	230
GM12878	1	2616
K562	1	1988

Supplementary Table S1 - Number of samples used in leave-one-out cross-validation on chromosomes 1 and 21 for cell lines GM12878 and K562 with window size 600nt.

Hi-C range	Hi-C_1L	Random_1L	Hi-C_2L	Random_2L	Hi-C_3L	Random_3L
10K	0.611	0.600	0.628	0.549	0.627	0.040
20K	0.626	0.624	0.613	0.372	0.614	0.032
30K	0.627	0.619	0.620	0.604	0.614	0.018
40K	0.612	0.619	0.623	0.614	0.615	0.265
50K	0.635	0.602	0.617	0.615	0.623	0.050

Supplementary Table S2 - The MCC scores for the same set up as in Table 5

Hi-C range	Hi-C_1L	Random_1L	Hi-C_2L	Random_2L	Hi-C_3L	Random_3L
10K	0.7946	0.795	0.777	0.6545	0.765	0.692
20K	0.7879	0.776	0.768	0.7117	0.796	0.682
30K	0.7934	0.773	0.768	0.766	0.798	0.712
40K	0.795	0.778	0.778	0.7668	0.777	0.699
50K	0.7926	0.770	0.778	0.7807	0.789	0.777

Supplementary Table S3 - The accuracy of the same SdA architectures as in Table 5 with pre-training epochs set to 10 and training epochs set to 10.

Hi-C range	Hi-C_1L	Random_1L	Hi-C_2L	Random_2L	Hi-C_3L	Random_3L
10K	0.530	0.439	0.501	0.040	0.474	0.008
20K	0.525	0.495	0.488	0.056	0.324	0.036
30K	0.531	0.505	0.480	0.425	0.357	0.008
40K	0.528	0.501	0.501	0.426	0.000	0.066
50K	0.534	0.499	0.510	0.376	0.196	0.000

Supplementary Table S4 - The MCC of the same configuration as in Table Supplementary Table S3.

Hi-C range	Hi-C_1L	Random_1L	Hi-C_2L	Random_2L	Hi-C_3L	Random_3L
10K	0.773	0.6903	0.763	0.639	0.765	0.642
20K	0.801	0.6994	0.768	0.641	0.776	0.759
30K	0.791	0.67	0.764	0.7156	0.771	0.662
40K	0.777	0.699	0.765	0.643	0.759	0.649
50K	0.795	0.739	0.795	0.634	0.763	0.625

Supplementary Table S5 - The accuracy of the same SdA architectures as in Table 5 with pre-training epochs set to 100 and training epochs set to 10.

Hi-C range	Hi-C_1L	Random_1L	Hi-C_2L	Random_2L	Hi-C_3L	Random_3L
10K	0.484	0.003	0.493	-0.003	0.495	-0.04
20K	0.375	0.044	0.508	-0.001	0.519	0.011
30K	0.392	0.026	0.502	0.017	0.507	0.015
40K	0.103	0.091	0.503	0.009	0.484	0.008
50K	0.276	0.027	0.499	-0.003	0.494	-0.005

Supplementary Table S6 - The MCC scores for the same configurations in Supplementary Table S5.

**Number of Processes**		P=1	P=2	P=4	P=8	P=16
Chr21	Time	703.05	370.69	201.41	99.97	56.58
	Speedup	-	1.89	3.49	7.03	12.43

Supplementary Table S7 - Execution time (seconds) and corresponding Speedup (time of using one process divided by the time using x processors, x = 2, 4, 6, and 16) on chromosome 21 of K562.
